# Is Exploration of Alternate Immune Pathways Needed in Hidradenitis Suppurativa? A Case of Atopic Dermatitis and Concurrent Hidradenitis Suppurativa Responding to Dupilumab

**DOI:** 10.1155/2023/5189034

**Published:** 2023-10-23

**Authors:** Sean McCormack, Noor Tazudeen, Benjamin C. Garden

**Affiliations:** ^1^Saint James School of Medicine, Cane Hall Road, Arnos Vale, Saint Vincent and the Grenadines; ^2^Private Practice, Chicago, IL, USA; ^3^University of Illinois at Chicago, Chicago, IL, USA

## Abstract

Hidradenitis suppurativa (HS) is an inflammatory dermatosis associated with overactive T helper 1/T helper 17 (Th1/Th17) cells. HS has been effectively treated with biologic medications; however, many such biologics lack large randomized controlled trials. Only one such biologic, adalimumab, has been approved by the US Food and Drug Administration (FDA) for the treatment of HS. Other such biologics currently being studied for HS downregulate Th1/Th17 inflammatory pathways. We describe a patient with atopic dermatitis (AD) and comorbid HS, both of which improved several months into treatment with dupilumab. Interestingly enough, dupilumab targets Th2-mediated inflammatory skin conditions through the inhibition of IL-4/IL-13 cytokines. While dupilumab is known for its success in treating Th2-mediated inflammation, this presents a paradox as HS is a Th1/Th17 inflammatory condition. This case highlights how the inflammatory process of HS is not fully understood and how biologic pharmacologic interventions need to be further studied to determine their efficacy in treating HS.

## 1. Introduction

Hidradenitis suppurativa (HS), also called acne inversa, is a chronic inflammatory condition involving the follicular portion of folliculopilosebaceous units (FPSUs). Symptoms of HS can be widely variable in severity. Features of HS include development of inflammatory nodules, sinus tracts, and fistulas with subsequent scarring around hair follicles. Commonly affected areas include intertriginous skin around the axillary, groin, perianal, perineal, and inframammary regions. Although HS more commonly occurs in these areas, HS can occur on any skin surface that contains FPSUs. The pathophysiology is not completely understood, but genetics, environment, and behavior all influence disease progression [1]. It is estimated that 1% to 4% of the population is affected, with women twice as likely to develop the disease compared to men [2]. Obesity, metabolic syndrome, and smoking are risk factors for the disease [3, 4]. Over time, the continued inflammatory response can destroy the pilosebaceous unit and adjacent structures [5, 6]. Clinical features associated with the lesions include pain and foul-smelling discharge which can be difficult to tolerate psychologically. The psychological impact due to decreased quality of life is often overlooked [6, 7].

HS is a disease that can be very difficult to treat. Many patients experience symptoms of this disease despite treatment. There are various treatment options with a spectrum of outcomes across patient populations [6]. Currently, there is only one US Food and Drug Administration- (FDA-) approved medication in the biologic category indicated for treatment. Adalimumab, a humanized anti-tumor-necrosis-factor-alpha (anti-TNF-*α*) monoclonal antibody, is approved for moderate-to-severe HS. Randomized clinical trials have shown the statistically significant benefits of adalimumab for treatment of HS [8, 9]. Another study has shown that 63.2% of patients achieve a clinical response by week 24. While this is great for the patients that do respond to treatment, there is still a large amount of patients who do not respond to available medications. Treatment failure is more common for patients diagnosed with HS compared to those with psoriasis, another chronic debilitating dermatological condition [9]. There is still a need for new treatments for HS or stronger supporting evidence to guide current therapy guidelines [10].

Herein, we describe a patient with a history of HS for many years that was started on dupilumab to treat concurrent atopic dermatitis (AD). While being treated with dupilumab for AD, it was observed that not only had the patient's AD improved but also the patient's HS had improved.

## 2. Case Report

A 28-year-old female former smoker with a history of morbid obesity, HS, and AD presented to the outpatient clinic for a follow-up on AD. The patient also has a history of psoriasis in the past, but this condition was not active. At the previous visit 3 months prior, the patient had been started on dupilumab injections and had been taking injections as prescribed (start: 600 mg divided in 2 sites x1; 300 mg subcutaneous injection every 2 weeks). Symptoms of AD had improved significantly. The patient was tolerating the medication well, and no side effects to the medication were reported. Dupilumab was her only current medication besides topicals as needed.

Physical examination revealed almost complete resolution of patches and plaques that had previously been distributed on her trunk and extremities. Over 10% body surface area (BSA) was previously affected. Other findings included rare nontender, noninflamed acneiform nodules distributed on the mons pubis, right axillary vault, and left axillary vault (Hurley stage 1).

On previous exams, the patient exhibited patches and plaques on the extremities and trunk, consistent with AD. The patient also exhibited psoriasiform plaques with micaceous scale on the right ulnar palm, left thenar eminence, right plantar midfoot, and left lateral midfoot; there was a concern that this may be pustular psoriasis. Most importantly, the patient had previously exhibited painful inflamed pustules and nodules with draining abscesses. These findings are seen in HS Hurley stage 2. Previous exams showed acute on chronic findings of HS, and the patient described the frequency of flares as consistently every few weeks.

The patient's AD, psoriasis, and HS had been refractory to many different treatments. Before presenting to the current practice, her previous psoriasis treatments included clobetasol ointment, triamcinolone cream, fluocinonide ointment, betamethasone ointment, roflumilast cream, adalimumab, methotrexate, risankizumab, ixekizumab, secukinumab, and guselkumab. Past attempted treatments for HS included topical antibacterial cleansers, mupirocin cream, clindamycin, doxycycline, metronidazole, rifampin, glycopyrrolate, and adalimumab. Treatments for AD included topical steroids and crisaborole ointment. The patient reported little benefit of many medications, insurance issues with many other medications, and a drug allergy to adalimumab. The patient had given up hope to find a solution to her HS.

Since starting dupilumab 3 months ago, the patient reported clearer skin. The patient had also noticed improved HS symptoms and no flares since starting the medication. The patient was satisfied with the improvements not only to her eczema but also to her HS. Due to current success, the treatment plan with dupilumab was continued at 300 mg subcutaneous injection every 2 weeks, and the patient was scheduled for follow-up.

6 months later at a follow-up visit, the patient reported that she was continuing to be doing well. Her symptoms of both AD and HS continued to be well controlled. She continued to experience zero flares of HS, regression of chronic lesions, and wound healing. Due to continued positive responses and toleration of the medication without side effects over the past 9 months, the treatment plan with dupilumab was continued at 300 mg subcutaneous injection every 2 weeks.

## 3. Discussion

The management of HS involves a multidisciplinary approach, including lifestyle modifications, topical and systemic medications, and surgery. Treatment outcomes with these topical and systemic medications and surgical interventions are variable, and each intervention comes with unique risks/benefits that must be individualized. Topical antiseptics and antibiotics can be used to manage mild-to-moderate cases of HS. For more severe cases, systemic treatments, such as oral antibiotics, and/or biologics, are often used. While antibiotics both topical and oral are used in the treatment of HS, it is important to distinguish that bacteria do not drive the pathogenesis of HS. Aspiration of unruptured lesions often results in a sterile culture, and patients are typically afebrile and lack other signs of infection. Antibiotics are used for their anti-inflammatory/immunomodulatory effects rather than their antibiotic properties. This includes actions such as suppression of various types of immune cells that would be otherwise contributing to inflammation around the FPSUs. Several guidelines have been formulated to direct clinical management of HS [1, 8, 10–12].

For moderate-to-severe disease, adalimumab is the only FDA-approved biologic. Adalimumab is a fully humanized anti-TNF-*α* monoclonal antibody and has been approved for treatment of HS since 2015. Two large RCTs (PIONEER I and PIONEER II) recruited 633 adults with moderate-to-severe HS. Patients were randomly assigned to receive either adalimumab or placebo in addition to topical antiseptic. Both studies showed that patients given adalimumab had greater reduction in the total number of abscesses and inflammatory nodules compared to placebo [9]. Our patient experienced an allergic reaction to adalimumab and subsequently desired different treatment options. Data behind other biologic treatments for HS are limited [8, 13–15].

Other biologic medications that may provide some benefit include infliximab, ustekinumab, anakinra, and secukinumab. The data supporting the efficacy of these drugs are not as robust [13–15]. In a RCT, infliximab (a chimeric anti-TNF-*α* monoclonal antibody) compared to placebo did not meet the primary endpoint (≥50% decrease in an unvalidated disease severity score); however, significant improvements in patient quality of life, pain, and physician global assessment scores were observed [16]. Interleukin-12/23 (IL) receptor antagonist ustekinumab has been studied in a small cohort, and 82% of patients had at least moderate improvement in symptoms [17]. IL-1 receptor antagonist anakinra has also been studied. Again, the sample size in the study was small; however, decreased disease activity was seen compared to placebo [18]. Secukinumab, an IL-17A receptor antagonist, has been shown to improve HS; however, data to show this are limited to case reports and a small pilot trial [19]. In summary, regardless of the biologic agent and its target, there are still not enough data on the effectiveness of these drugs. Currently available studies are not extensive enough to evaluate the efficacy of these biologics on a larger scale. As these drugs become more accessible over time, it is likely that more information on their efficacy becomes available. Previous authors have published in-depth literature reviews of currently studied biologics and their respective diseases including but not limited to HS [13–15].

Searching the PubMed database revealed only two case letters on the successful use of dupilumab in a patient with AD and concomitant HS [20, 21]. Many other biologics are being studied for the treatment of HS; however, it seems that dupilumab is not one of them. This could be due to the fact that most usually consider HS mainly as a Th1/Th17-mediated inflammatory disease. Multiple studies have shown the association of Th1/Th17-associated cytokines including TNF, IFN*γ*, IL-10, IL-12, IL-17, IL-23, IL-32, antimicrobial peptides (AMP), LL-37, Psoriasin, and *β*-defensins (hBD) 2 and 3 around the lesional inflammation seen in HS [14, 22]. Dupilumab is a biologic medication that inhibits IL-4/13 cytokines involved in Th2-mediated inflammation. AD is mainly a Th2-mediated inflammatory disease [23]. However, patients with a diagnosis of AD have an approximately 5.57-fold increased odds ratio of having HS as compared with those who do not have AD [24]. This is interesting as Th1/Th2 immune pathways exert opposing actions along the immune axis. There may be more immunological pathways implicated in HS than what we are currently aware of.

Modern theories agree that follicular occlusion is likely responsible for the initial development of the condition. Follicular occlusion precedes follicular rupture with subsequent inflammatory responses and formation of abscesses, sinus tracts, and fistulas. Over time, the continued inflammatory response can destroy the pilosebaceous unit and adjacent structures [5, 6]. While we may understand how the disease progresses, there may be more complex immunological processes involved in driving the disease than currently studied.

The authors that have presented two cases similar to ours have described possible mechanisms of immune dysregulation that may play a role in HS. These include genetic susceptibility, notch signaling dysregulation, epidermal barrier defect, AMP dysfunction, and alteration of sphingolipid metabolism [20, 21].

In one of the two case reports, the patient had a history of psoriasis [20]. This is similar to the patient we present. It is uncommon to see patients who exhibit concurrent findings of psoriasis and AD. Others have hypothesized that these patients may represent a unique population with possible underlying genetic predisposition. It has also been hypothesized that inhibition of a specific T cell pathway through medications can result in an immunologic axis imbalance [25].

## 4. Conclusion

HS is a chronic condition that severely affects the quality of life for those diseased. While there are a variety of different treatment options, HS tends to be refractory to treatment, and response is often poor. More biologics need to be studied, as there may be multiple immune pathways implicated in disease progression. For treatment to be effective, we may need to target more than one pathway. We report an unusual case in which dupilumab was used to treat AD in a patient with HS. Several months into the treatment with dupilumab, the patient reported improved AD but also improved HS. Dupilumab has shown success in two other cases: AD and concomitant moderate-to-severe HS [21, 22]. Dupilumab, a biologic medication that inhibits IL-4/13 cytokines involved in Th2-mediated inflammation, may be of use in the treatment of HS. Further investigation into the effects of targeted biologics for HS including dupilumab is needed.

## Data Availability

No data were used to support this study.
